# Foreign Body in the Vagina

**Published:** 1901-11

**Authors:** Carroll W. Downey

**Affiliations:** Rutherfordton, N. C.


### FOREIGN BODY IN THE VAGINA.

By CARROLL W. DOWNEY, M.D.,

Rutherfordton, N. C.

I was recently called to see a patient, Miss M., who gave the history of having been treated at intervals for two years for an endometritis, the treatment being such as we are accustomed to give by the mouth, uterine tonics and the like. The treatment had been without result. No local treatment of any kind had been carried out. I told her that I would have to make a vaginal examination and treat her locally, but she declined to submit to the examination and dismissed me as her physician, exacting a promise from me, however, that I would return if she did not improve and if she decided to send for me. During the following night she suffered so intensely that the attending physician and her family decided that she had appendicitis and I was again called in at an early hour the next morning. To my surprise I found the patient anxious for an examination. On digital exploration I discovered a tumor mass in the vaginal wall, very sensitive to the touch. Not being satisfied with the digital examination I introduced a speculum and found that the mass had the appearance of a furuncle or carbuncle. Although the manipulations were very painful I decided to draw down the womb so that a better view might be obtained. This was done by traction on the cervix, and a small hard mass was seen in the center of the swelling. Incision at this point liberated a foreign body followed by a gush of pus. The foreign body proved to be a piece of pine bark $1\frac{1}{2}$ in. long and $\frac{1}{2}$ in. wide. Quite a little cavity was left which did not, however, extend through the thickness of the vaginal wall. The cavity was washed with bichloride solution and packed with iodoform gauze. The patient made a rapid recovery. As soon as the foreign body was removed all pain vanished and the patient was able to do manual labor in a short time. The monthly periods were regular and easy thereafter. The patient offered no explanation of the presence of the bark in the vagina.

